# The Importance of Dental Service in the Hospital

**Published:** 1919-07

**Authors:** Alfred A. Crocker

**Affiliations:** Cincinnati, Ohio


### THE IMPORTANCE OF DENTAL SERVICE IN THE HOSPITAL

### Importance in Diagnosis and Systemic Disease—Possibilities of Scientific Research in Dental Department—Large Field of Operation Found Among the Children

BY ALFRED A. CROCKER, CINCINNATI, OHIO

Dental service is necessary along with medical service in healing systemic disease. The relationship of the teeth to systemic diseases as foci of infection, or at least as relay posts from which the infection is increased, has so repeatedly been demonstrated that most physicians recognize it as a valuable element in their diagnosis. The examination of the mouth condition of the patient furnishes valuable data in determining the treatment of the patient while in the hospital, as it tells whether, owing to the presence of pyorrhea, alveolar abscess, blind abscess, impacted unerupted teeth, the teeth can be counted in the treatment to follow, or eliminated from consideration. Work for the dental department of a hospital is furnished by maternity cases, children's teeth, and cases in which arsenic, iodides, mercury or phosphorus are prescribed. Facilities for difficult extractions under anesthesia and for scientific research are also afforded by a hospital dental clinic.

During a recent visit to the Jewish Hospital in Cincinnati, where they have a dental clinic with Dr. Samuel Rabkin in full-time attendance as dental clinician, I was shown the hospital card on which the tooth condition of all patients is recorded by him. All his findings are recorded thereon and furnish data which very often help the hospital board, together with the records placed on the same card by physicians and specialists in the hospital's other departments, in arriving at the correct diagnosis and subsequent relief and restoration of the patient to full health and usefulness. As Dr. Hexter, the supervisor, explained, it is not enough to place the patient on his feet; to restore the patient to his or her normal self, if possible, is the aim of the hospital, and it takes an analysis from all departments to do it properly. Another work performed by the dental clinic at the Jewish Hospital is the care of children's teeth, the advantages of which all welfare authorities readily recognize. This work is part of the oral hygiene movement which is so active in all parts of the United States.

Besides the hospital dental work above outlined, research work in dental pathology and dental bacteriology is carried on in connection with cases presented at the hospital. This work has shown valuable results and has been of much benefit to the patients treated at the hospital.

A visit was also made at the Cincinnati General Hospital, where Dr. W. S. Locke has charge of the dental department. Patients arriving at the hospital for diagnosis and treatment are taken to the dental clinic at the direction of the attending physician. If the patient is unable to walk, a wheeled chair or a wheeled stretcher is used to convey the patient there. After the instrumental examination of the teeth, the dental clinician sends the patient to the X-Ray department for a complete dental roentgenographic examination. Ten films are taken, five upper and five lower, covering the complete mouth. One of the large machines is used on a two and one-half spark gap. The developed films are returned to the dental department and, together with the clinical report thereon, sent to the attending physician with the dental recommendations for the case. The dental diagnosis, along with the attending phsyician's diagnosis of symptoms and other tests, such as urinalysis, bacteriological, etc., make up the case history, which is kept on an indexed chart or card. In all cases the clearing up of mouth conditions accelerates recovery from the systemic conditions. Part of every day is devoted to the care of children's teeth at the Cincinnati Hospital. Children from the city and neighborhood are brought to the hospital. Care of their teeth is along preventive lines and correction of irregularities and dental advice to the parents. Children from five years up receive this attention and learn the value of care of the teeth.

Oral surgery is performed for all patients coming to the hospital, who receive immediate attention by the skilled operatives in the dental department.

Dentistry in the hospital has shown its value in so many ways and so many patients arriving at the hospital require dental care that the department is very much in demand.—*Modern Hospital* for May.

